# Whole-Cell Fiber-Optic Biosensor for Real-Time, On-Site Sediment and Water Toxicity Assessment: Applications at Contaminated Sites Across Israel

**DOI:** 10.3390/bios15070404

**Published:** 2025-06-22

**Authors:** Gal Carmeli, Abraham Abbey Paul, Kathelina Kristollari, Evgeni Eltzov, Albert Batushansky, Robert S. Marks

**Affiliations:** 1 Avram and Stella Goldstein-Goren Department of Biotechnology Engineering, Ben-Gurion University of the Negev, Be’er-Sheva 84105, Israel; galca@post.bgu.ac.il (G.C.); paulab@post.bgu.ac.il (A.A.P.); kathelin@post.bgu.ac.il (K.K.); 2 Institute of Postharvest and Food Science, Department of Postharvest Science, Volcani Center, Agricultural Research Organization, Rishon LeZion 7505101, Israel; eltzov@volcani.agri.gov.il; 3 Agro-Nanotechnology and Advanced Materials Research Center, Volcani Institute, Agricultural Research Organization, Rishon LeZion 7505101, Israel; 4 The Ilse Katz Institute for Nanoscale Science and Technology, Ben-Gurion University of the Negev, Be’er Sheva 84105, Israel; albertbat@bgu.ac.il

**Keywords:** bioreporter, bioluminescent bacteria, optical-fiber biosensor, water toxicity, sediment toxicity, in situ monitoring, whole-cell biosensor

## Abstract

Sediments are key players in the optimum functioning of ecosystems; however, they also represent the largest known repository of harmful contaminants. The vast variety of these sediment-associated contaminants may exert harmful effects on marine communities and can impair ecosystem functioning. Whole-cell biosensors are a rapid and biologically relevant tool for assessing environmental toxicity. Therefore, in this study, we developed a bioassay-based toxicity measurement system using genetically modified bacteria to create a whole-cell optical biosensor. Briefly, reporter bacteria were integrated and immobilized using a calcium alginate matrix on fiber-optic tips connected to a photon counter placed inside a light-proof, portable case. The calcium alginate matrix acts as a semi-permeable membrane that protects the reporter-encapsulated optical fiber tips and allows the inward passage of toxicant(s) to induce a dose-dependent response in the bioreporter. The samples were tested by directly submerging the fiber tip with immobilized bacteria into vials containing either water or suspended sediment samples, and the subsequent bioluminescent responses were acquired. In addition to bioavailable sediment toxicity assessments, conventional chemical methods, such as liquid chromatography–mass spectroscopy (LC-MS) and inductively coupled plasma optical emission spectroscopy (ICP-OES), were used for comprehensive evaluation. The results demonstrated the efficacy of the biosensor in detecting various toxicity levels corresponding to identified contaminants, highlighting its potential integration into environmental monitoring frameworks for enhanced sediment and water quality assessments. Despite its utility, this study notes the system’s operational challenges in field conditions, recommending future enhancements for improved portability and usability in remote locations.

## 1. Introduction

Sediments are key players in the functioning of aquatic ecosystems as they directly impact the lives of benthic organisms that occupy a critical stage in complex aquatic food relationships. The toxicity evaluation of water and sediments that may impact marine life has continued to attract research efforts [1]. In particular, sediments are known to be the largest chemical repositories on Earth, where a vast variety of harmful, often persistent, hydrophobic compounds accrue and are retained long after the pollution of the overlying water has decreased or become undetectable [2]. Tests that can provide valuable information about the bioavailability and combined effects of multiple pollutants present in the environment are desirable [1]. There is a growing body of evidence suggesting the direct harmful effects of a variety of sediment-associated contaminants on the optimal functioning of an ecosystem [2,3]; however, environmental authorities such as the European Union Water Framework Directive (EU-WFD) place more emphasis on water toxicity monitoring than sediment toxicity in assessing the health of aquatic ecosystems [4]. Moreover, in events when sediment toxicity analyses are conducted, monitoring authorities usually assess the sediment quality through targeted chemical analysis, concentrating on a limited range of specific compounds and with little or no emphasis on the ecotoxicological risks that arise from the numerous (un)known mixtures of sediment-associated compounds [5]. There is a need for methods that can account not only for the presence of contaminants, but also for the biological effects of contaminants on marine biota. Traditional analytical methods such as gas chromatography (GC), liquid chromatography (HPLC), and inductively coupled plasma mass spectrometry (ICP-MS) are precise but time-consuming, expensive, and often inadequate for the precise evaluation of the bioavailability and biological effects of contaminants. Moreover, these methods typically focus on chemical identification and quantification without directly addressing biological toxicity [6,7]. By using bioassays to evaluate toxicity to various organisms, such as amphipods, midges, and bivalves, the ecological risks posed by contaminated water and sediments can be determined [8]. Sediment toxicity tests are bioassays in which benthic organisms are exposed to field-contaminated or spiked sediment, and their survival, growth, emergence, and/or reproduction are generally evaluated after a certain exposure duration [2]. Specialized techniques, such as direct spiking and passive sampling, are being used to obtain more accurate and reliable toxicity assessments [9].

Whole-cell biosensing systems possess attributes that make them ideal for portable field kits. These attributes include the ability to withstand a wide range of environmental conditions (such as temperature, pH, and ionic strength), providing information on analyte bioavailability, requiring minimal or no sample pretreatment, high sensitivity and selectivity, easy preparation, rapid detection, cost-effectiveness, high-throughput screening, and miniaturization [10]. These biosensors are based on genetically engineered microorganisms (broadly referred to as bioreporters) that produce quantifiable signals such as bioluminescence and fluorescence in response to specific toxicants or environmental stressors [11]. The immobilization of bioreporters for the development of fiber-optic-based biosensors has proven to be an indispensable bioanalytical approach for effect-based on-site and continuous assessment of water and sediment toxicity [12]. Whole-cell fiber-optic biosensors have demonstrated their effectiveness in monitoring a variety of contaminants, including genotoxicants [13], heavy metals [6], organic pollutants [11], and specific toxic events, such as those observed in Lachish River contamination [14]. It is believed that by integrating effect-based methods with chemical profiling, these tests provide a comprehensive understanding of the potential risks associated with contaminated aquatic environments [1].

**Figure 1:**
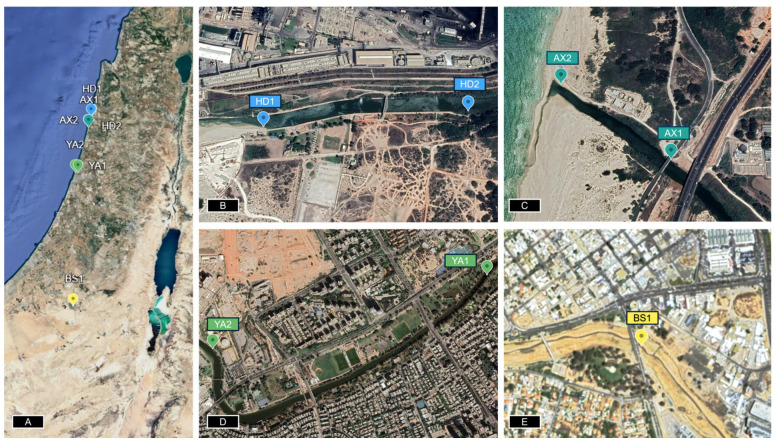
Sample collection. (**A**) Sampling sites across Israel; (**B**) Hadera (HD2) Stream; (**C**) Alexander (AX1) River; (**D**) Yarkon (YA2) River; and (**E**) Beersheba (BS1) stream. The coordinates are listed in Supplementary Table S1).

**Figure 2:**
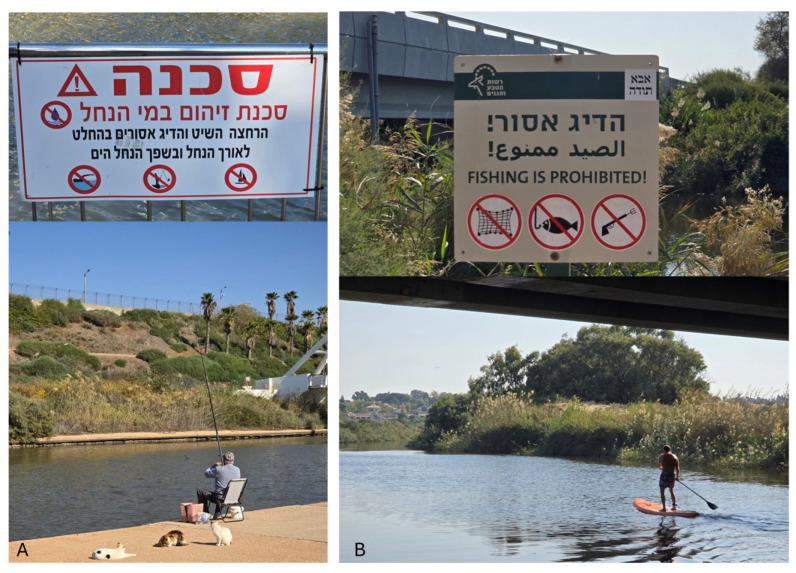
Human activity at potentially polluted sites. (**A**) Fishing at Hadera Stream alongside a sign that says “Danger—risk of contamination, bathing, sailing, and fishing is strictly prohibited”; (**B**) sailing at Alexander River.

The goal of this study was to develop a field-enabled whole-cell optical biosensor for on-site direct sediment toxicity measurement using a genetically modified *Escherichia coli* strain expressing bioluminescent reporter genes (luxCDABE) under the control of promoters responsive to general stress that induces the expression of heat shock protein families [15]. The reporter bacterial strain was encapsulated onto the near end of an optical fiber using a biocompatible calcium alginate hydrogel matrix. The hydrogel matrix served as a semi-permeable membrane that retained the bacteria and selectively allowed the passage of toxicants into the bioreporter, triggering a dose-dependent bioluminescence response. Water and sediment samples from multiple potentially contaminated sites across Israel (Figure 1 and Figure 2) were evaluated for direct water and sediment toxicity and chemical analyses. The bioreporter-immobilized optical fiber was used for both direct (in situ) bioavailable sediment toxicity and sediment extracts (Supplementary Material Figure S2). Because of the complex matrix, microplate reader analysis and LC-MS analysis were conducted only on the water samples and extracts, while the fiber-optic set enabled a comparable toxicity measurement at the point of site. The present study demonstrates the practicality of direct sediment toxicity measurements without the need for complex sample preparation steps.

## 2. Materials and Methods

### 2.1. Materials

Calcium chloride, ampicillin (A9518-5G), and low-viscosity sodium alginate (A-2158), were purchased from Sigma-Aldrich (Saint Louis, MO, USA), Luria-Bertani medium (LB broth), and LB agar Difco (244520) were purchased from Becton (Dickinson & Company, Le Pont de Claix, France), and ethanol (19-009101-80, Romical, Israel) were of analytical grade.

### 2.2. Sample Collection

Water and sediment samples were collected from the banks of potentially contaminated streams and rivers across Israel [16,17,18]. Soil samples were collected from four locations across the northern and southern regions of Israel in September and October 2024 (Figure 1) from the top 10 cm layer. Water samples were collected by submerging a 50 mL tube into the water body, while sediment samples were collected using a 0.5 L plastic container and stored refrigerated until required.

### 2.3. Bacterial Toxicity Measurement

**Figure 3:**
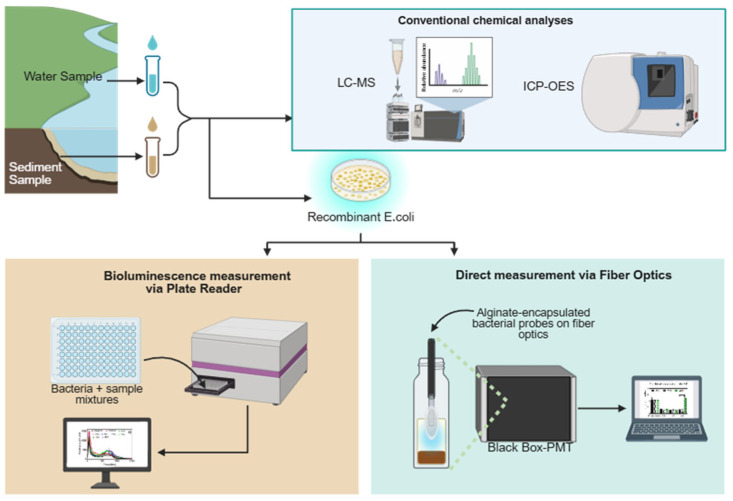
Experimental workflow for this study. Water and sediment samples were evaluated using both a plate reader and a field-enabled optical fiber whole-cell biosensor in combination with conventional methods (Created in BioRender. Marks, R. (2025), https://BioRender.com/9yr6jqi, accessed on 20 February 2025).

Two bacterial toxicity measurement approaches were employed: direct bioavailable sediment toxicity testing and the testing of toxicants extracted from the sediment. Laboratory-based testing was conducted on extracted bioavailable sediment and water samples. Finally, conventional chemical analyses were performed to provide more insight into the chemical nature of the sample, as shown in the experimental workflow (Figure 3).

#### 2.3.1. Strain Description

**Figure 4:**
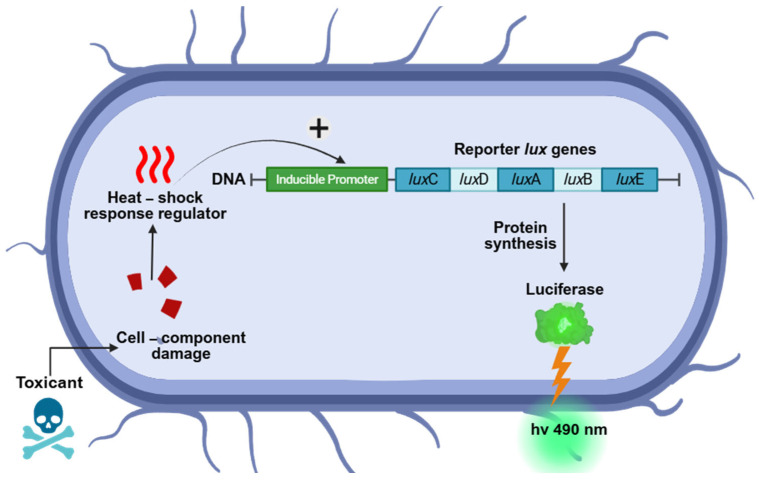
Mechanism of bioluminescence toxicity measurements. A toxicant penetrates the cell wall and binds to an intracellular heat shock receptor, initiating a signaling cascade to the grpE promoter. This promoter facilitates the transcription of the reporter gene (luxCDABE), leading to the induction of lux gene expression. Consequently, luciferase synthesis increases, resulting in a detectable luminescence signal. Toxicants cause protein damage, which activates specialized regulatory proteins that simultaneously trigger stress response genes and lux genes. When the stimulus is sufficiently harmful to impair cellular function and disrupt metabolic processes, luminescence decreases, owing to reduced luciferase synthesis and substrate depletion (created with biorender.com, accessed on 20 February 2025).

The organism chosen to demonstrate the proposed system’s applications and used in all the experiments was the *E. coli* strain TV1061 [19]. This strain contains a plasmid-based fusion of the *E. coli* grpE promoter with the *Photorhabdus luminescens* luxCDABE reporter operon. The lux operon consists of five structural genes that encode the heterodimeric luciferase enzyme (*lux*A and *lux*B) and the biosynthetic enzymes (luxC, *lux*D, and *lux*E) responsible for producing the luciferase substrate, tetradecanal, in an ATP- and NADPH-dependent manner [20]. The grpE promoter is part of the heat shock response system, which is activated under conditions of cellular stress and metabolic changes, such as cytotoxic substances, as shown in Figure 4 [19,21,22]. Studies have demonstrated that in liquid culture, *E. coli* TV1061 responds to various cytotoxic stressors, including ethanol, heavy metals, and heat-shock-inducing compounds, by producing a dose-dependent increase in bioluminescence at 490 nm [13,14,23,24]. This response renders TV1061 a valuable bioreporter strain for detecting general cytotoxicity and environmental stressors.

#### 2.3.2. Culturing Conditions

The bacteria were cultivated on Luria–Bertani (LB) agar supplemented with ampicillin. Cultured plates containing 100 µg/mL ampicillin were incubated at 37 °C for 24 h and then stored at 4 °C for further use. The cultures maintained their plasmid replication for up to 30 days, after which they were refreshed. Prior to sample analysis, one bacterial colony from the pure culture was grown in 10 mL of Luria–Bertani (LB) broth supplemented with the appropriate antibiotic for 24 h at 37 °C with shaking at 220 RPM (TOU-120 BenchTop Orbital Shaking Incubator, MRC Ltd., Holon, Israel). A secondary culture was prepared for each experiment by growing bacteria in a fresh 30 mL antibiotic-free LB medium (1:50 dilution) for 2–3 h at 37 °C with shaking at 220 RPM (TOU-120 BenchTop Orbital Shaking Incubator, MRC Ltd., Holon, Israel). The bacterial concentration was monitored using a spectrophotometer at a wavelength of 600 nm (Ultrospec 2100 Pro, Amersham, UK). Cultures reaching an opti cal density at 600 nm (OD_600_) of 0.2–0.3 (approximately $10^{6}$ cfu/mL) were suitable for the assay since they are the most active at this stage. This specific stage within the exponential growth phase is crucial to avoid the need for dilution during measurement.

#### 2.3.3. Preparation of Bacterial Suspensions for Immobilization

The 30 mL tubes containing a fast-growing reporter bacterial strain (*E. coli* TV1061) culture with an OD_600_ of 0.6–0.8 (approximately 3 × $10^{6}$ CFU/mL) were centrifuged at 4000 rpm for 10 min. The top 25 mL of the centrifuged suspension was discarded, and the cells were resuspended in the remaining 5 mL, resulting in an OD_600_ of approximately 1.2–1.4. The harvested cells were mixed with filter-sterilized 2.5% (*w*/*v*) sodium alginate solution at a volume ratio of 1:4 (bacteria: alginate).

#### 2.3.4. Sample Collection and Preparation for Analysis

Water samples were filtered using a 45 µm syringe filter and tested on-site and in the laboratory using a microplate reader (BioTek Synergy H1, Santa Clara, CA, USA). The sediment samples were dried in an oven (BINDER GmbH, Tuttlingen, Germany) at 60 °C for 48 h. The sediments were weighed before and after drying to calculate their moisture contents (Supplementary Table S2).

**Figure 5:**
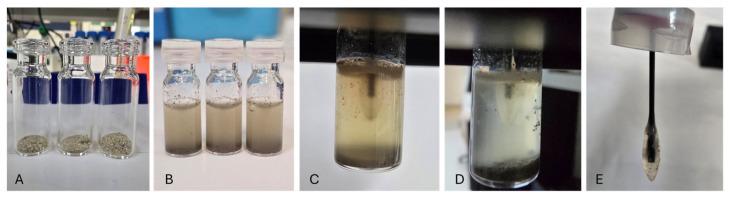
Direct testing using the fiber-optic whole-cell bacterial setup (black box). (**A**) Dry sediment samples; (**B**) sediment samples in LB broth and CaCl_2_; (**C**) probe immersed in solution B prior to measurements; (**D**) sample following measurement; (**E**) probe outside of the vial after measurements. The appearance of the biosensor probe during water measurement is shown in Figure S1 in the Supplementary Material.

In the case of sediment toxicity assessment, two approaches were employed: preparation of bioavailable toxicants from the sediment and direct sediment toxicity measurement (Figure 5). For bioavailable toxicity measurements, all dried sediment samples were mixed with DDW at a soil/sediment: water (*w*/*v*) ratio of 1:5 [6]. The obtained suspensions were vortexed for 1 min and then either passed through a 45 µm syringe filter or left to extract for 24 h before being filtered through a 45 µm syringe filter and then refrigerated until required. Meanwhile, for direct sediment toxicity measurement, a determined amount (114.1 ± 2.78 mg of dry, or 132.77 ± 29.27 mg of wet) of sediment samples per milliliter assay media was prepared and used for direct fiber-optic-based toxicity measurement. In all experiments, a 1% (*v*/*v*) solution of ethanol was used as a standard inducer of the *E. coli* (TV1061) bioreporter strain [14,23], and sterile DDW served as the negative control.

#### 2.3.5. Bioluminescence Measurement Using Multimode Plate Reader

The bioluminescent toxicity responses of the bioreporter TV1061 strain, as well as the optical density at 600 nm (OD_600_), were acquired over a 10–24 h period using a BioTek Synergy H1 microplate reader (Agilent, Santa Clara, CA, USA) and maintained at 37 °C. The luminometer was set to kinetic mode at 15 min intervals between readings. Either white, (non-)transparent, 96-well, flat-bottom microplates (Nunc, Roskilde, Denmark) or white, 96-well, non-binding, µClear^®^ microplates (Greiner Bio-One GmbH, Kremsmünster, Austria) were used in all experiments. Experiments were performed in three or four biological replicates. The maximum luminescence values (expressed in the relative luminescent unit, RLU) were extracted using Microsoft Office Excel, from which the induction factor was calculated as a ratio of the maximum RLU of the test to that of the negative control (in the absence of an inducer) [25].

(1) $$Induction\;Factor\;\left(I.F\right)=\frac{(RLU/OD600)\;test}{(RLU/OD600)\;non-induced\;control}$$

A calibration curve was generated by preparing serial dilutions (1.0-, 0.9-, 0.75-, 0.6-, 0.4-, and 0.1-fold) of sediment and water samples in sterile double-distilled water (DDW), where 1.0 represents the undiluted sample and 0.1 the most diluted. The corresponding bioluminescent responses were measured as previously described.

#### 2.3.6. Immobilization of the Bioluminescent Bacteria onto the Optical Fiber

**Figure 6:**
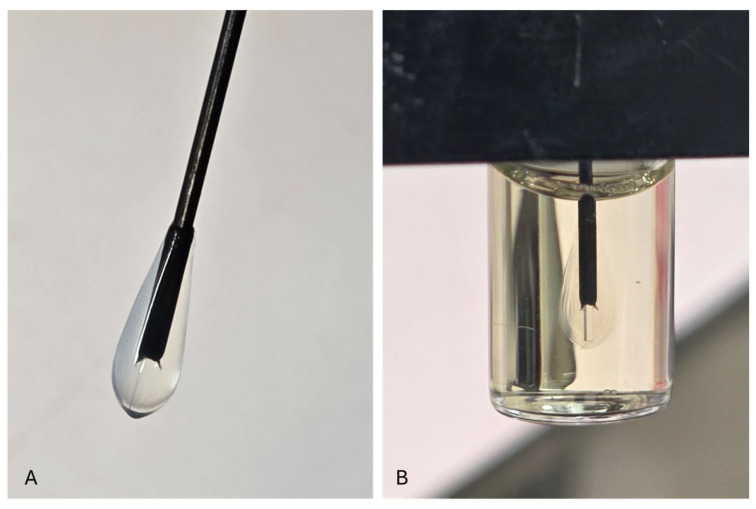
Setup of fiber-optic probe: (**A**) encapsulated in alginate bacteria and (**B**) inserted into the black box housing the bacterial probe and photon-counting unit of the whole-cell biosensor unit.

The optical fiber used was SFS400/440, Molex (Fiberguide, Lisle, IL, USA), a multimode fiber with a core diameter of 400 µm, cladding diameter of 440 µm, numerical aperture (NA) of 0.22 ± 0.02, and wavelength transmission range of 190–1250 nm. The core material was pure fused silica, and the cladding was fluorine-doped silica with refractive indices of 1.457 (core) and 1.440 (cladding) at 633 nm. Prior to preparation, black nylon jackets (cladding) covering the fibers were stripped from a 1 cm long optical fiber tip. The stripped fiber tips were washed with a 70% (*w*/*v*) ethanol solution before immobilization. The 1 cm optical fiber tip was dipped (for a few seconds) into the bacteria–alginate suspension and then (for a few seconds) into a sterile 0.15 M calcium chloride solution. This procedure resulted in the formation of a solid Ca alginate matrix that attached alginate-entrapped bacteria to the fiber [25]. The calcium alginate hydrogel matrix is optically transparent across the visible spectrum, with minimal light scattering or absorption at the bioluminescence emission peak (~490 nm). Its refractive index typically ranges from 1.334 to 1.345 [26] depending on the alginate concentration and crosslinking density, which closely matches that of water and ensures efficient light transmission to the fiber core. This procedure was repeated six times to increase the number of bacterial sensor cells attached to the optical fiber transducer [6,13,24,27]. Fiber-optic sensors were used immediately after the preparation (Figure 6). After each measurement, the alginate layer was removed and the optical fiber was sterilized by immersion in 70% (*w*/*v*) ethanol, allowing the fiber to be reused for subsequent probe preparation.

#### 2.3.7. Fiber-Optic Instrument (Black Box) Setup

The “fiber-optic black box” refers to a custom-built, light-tight enclosure designed to eliminate ambient light interference and maintain a stable internal temperature during bioluminescence detection using the photon-counting system.

The photon-counting system was designed and built in our laboratory, as described recently [25]. The instrument setup was placed in a light-tight box to prevent environmental light interference. A manual shutter (71430, Oriel, Franklin, MA, USA) was placed in front of the detector to protect the photon-counting unit and was operated externally using a workshop-made lever. The output signal in the analog measurements was the mean value of the pulses generated after the multi-anode amplification in the photomultiplier tube. A P25PC Photodetector Module (Sens-Tech Ltd., Sawston, Cambridge, UK) featuring a 25 mm end-window photomultiplier tube with a blue-green sensitive S20 photocathode was employed for bioluminescence detection. The detector operates with low dark counts, high-speed amplification, and magnetic shielding, thereby ensuring enhanced sensitivity in the blue-light spectral range. The module’s 22 mm active diameter allows for effective light collection without requiring additional optical focusing elements. Data acquisition was performed using Counter/Timer software provided by Sens-Tech Ltd. (Version 2.8, Build: 11), enabling precise photon counting and real-time analysis. A workshop-made fiber holder was used to secure the fiber in front of the detector. To support bacterial viability and maintain a stable temperature, a 15.5-by-22.0 cm^2^, 5 V 2 A USB-powered, 7.5 W commercial reptile heating mat was placed inside the black box and was set to 33 °C ± 3 during measurements. This temperature was selected to provide a physiologically optimal environment for *E. coli*, which demonstrates stable growth and luciferase expression within the 25–37 °C range [28]. Maintaining this steady temperature minimizes the variability in bacterial metabolism and reporter activity, particularly during extended (up to 24 h) measurements, and ensures reproducibility across experiments conducted under both laboratory and field conditions. To prevent ambient light from interfering with photon detection, the entire biosensor system was enclosed in a custom-built light-tight box. This enclosure eliminated external light contamination during bioluminescence acquisition, which is particularly important because of the transparency of the calcium alginate matrix. The optical fiber was securely positioned in front of the photomultiplier tube (PMT) using a fixed fiber holder, ensuring that only the light emitted by the encapsulated bacteria at the fiber tip reached the detector. This optical isolation, combined with precise fiber–detector alignment, ensured high signal fidelity and excluded environmental photon interference.

#### 2.3.8. Fiber-Optic Bioluminescent Toxicity Acquisition

The fiber tips containing the adlayers of alginate-encapsulated bacterial probes were submerged into vials (1.5 mL Snap-Cap Vial, AIJIREN Technology Co., Ltd., Quzhou, China) containing 900 µL of Luria–Bertani (LB) Broth Difco (244629) medium, 10 µL of sterile 0.15 M calcium chloride solution, and 100 µL of the tested sample (either extracted bioavailable sediment toxicants or water samples). Optical fibers were passed through a small hole in the vial cap created using a 16-gauge sterile needle. The vials were placed inside a photon-counting biosensor system in a plastic tray (AIJIREN Technology Co., Ltd., Quzhou, China) using a heating mat. The other end of the fiber was secured in front of the PMT, and the bioluminescence response was measured for up to 24 h. For the direct sediment toxicity testing, 114.1 ± 2.78 mg of dry sediment or 132.77 ± 29.27 mg of wet sediment samples were added to one milliliter of assay media containing 10 µL of sterile 0.15 M calcium chloride solution, and the bioluminescent responses were acquired as described for the water or extracted sediment samples. The representative bioluminescent kinetic curves are shown in the Supplementary Materials (Figure S3)

Complementary chemical analyses were performed using inductively coupled plasma optical emission spectroscopy (ICP-OES) and liquid chromatography–mass spectrometry (LC-MS).

### 2.4. Conventional Chemical Analyses

#### 2.4.1. Elemental Analysis by ICP-OES

Heavy-metal concentrations in the collected environmental water samples were determined using inductively coupled plasma optical emission spectroscopy (ICP-OES) (SPECTROGREEN ICP-OES, SPECTRO Analytical Instruments GmbH, Kleve, Germany). The instrument was operated with dual viewing plasma modes—axial end-on plasma (EOP) and radial side-on plasma (SOP)—to enhance sensitivity and precision. Sample introduction was performed using a crossflow nebulizer at a rate of 2 mL/min. Calibration standards covering a concentration range from 0.05 ppm to 100 ppm were prepared from certified stock solutions to quantify metals, including Ag, Al, Ba, Cd, Co, Cr, Cu, Fe, Mn, Ni, Pb, Sr, Ti, Zn, and others, along with separate phosphate and sulfate calibration sets. The emission lines for each element were carefully selected based on optimal sensitivity and minimal spectral interference. Quality control procedures included blank measurements, standard checks, and polynomial regression analysis to confirm instrument linearity (minimum correlation coefficient > 0.996) and accuracy within the specified analytical ranges.

#### 2.4.2. LC-MS Analysis

Non-Targeted Profiling (NTP) was performed using an LC-MS system (liquid chromatography coupled with high-resolution mass spectrometry). The extracted sediment and water samples were microfiltered, and 200 µL of the filtrate was completely dried in a vacuum concentrator. The dry pellets were resuspended in 50 µL of LC-MS-grade acetonitrile and 10 µL of internal standard (^13^C_3_-cortisone 100 µg/mL in methanol), shaken for 1 min, and centrifuged twice at maximum speed for 10 min to remove insoluble materials. The pure liquid was transferred into an LC vial, and 1 µL was injected into the LC-MS system consisting of a Waters Acquity Liquid Chromatograph (Waters, Milford, MA, USA) equipped with an HSS-T3 reverse-phase column (Waters Corp, Milford, MA, USA) and a Thermo Fisher Scientific (Waltham, MA, USA) Q Exactive Plus Mass Spectrometer fitted with an electron spray ionization source. A gradient of mobile phases A (0.1% formic acid in water) and B (0.1% formic acid in acetonitrile) was used in the following order: 0–1 min 99% of A, 1–11 min 60% of A, 11-13 min 30% of A, 13-15 min 1% of A, 15-16 min 1% of A, 16–17 min 99% of A, and 17–20 min 99% of A. The flow rate was 0.4 mL/min, and the temperature of the column was 40 °C [29]. Data acquisition was performed in the full-scan (60–1000 *m*/*z*) positive ionization mode. In addition, the blank sample was analyzed to remove background data, and the quality control pool was analyzed in both full-scan and MS/MS modes for secondary fragmentation data. Peak extraction and background filtering were performed using Thermo Fisher Scientific Compound Discoverer software, version 3.3. Putative annotation of metabolites was performed in Compound Discoverer by matching the detected high-resolution mass spectrum to the ChemSpider public database and the MS2 spectrum (where available) to the Thermo Fisher Scientific mzCloud database. Downstream data analysis was performed using Microsoft Excel (Version 2505, Build 18825.20150) and R-project software (R version 4.3.3) [30]. A heatmap was built using the “pheatmap” package (version 1.0.13) [31].

### 2.5. Statistical Analysis

A two-way or one-way ANOVA, followed by a Tukey multiple-comparisons test, was performed using GraphPad Prism version 8.0.2 for Windows (GraphPad Software, San Diego, CA, USA, www.graphpad.com, accessed on 9 November 2024).

## 3. Results

### 3.1. Bioluminescent Toxicity Response

#### 3.1.1. The Fiber-Optic-Based Bacterial Toxicity Measurement

**Figure 7:**
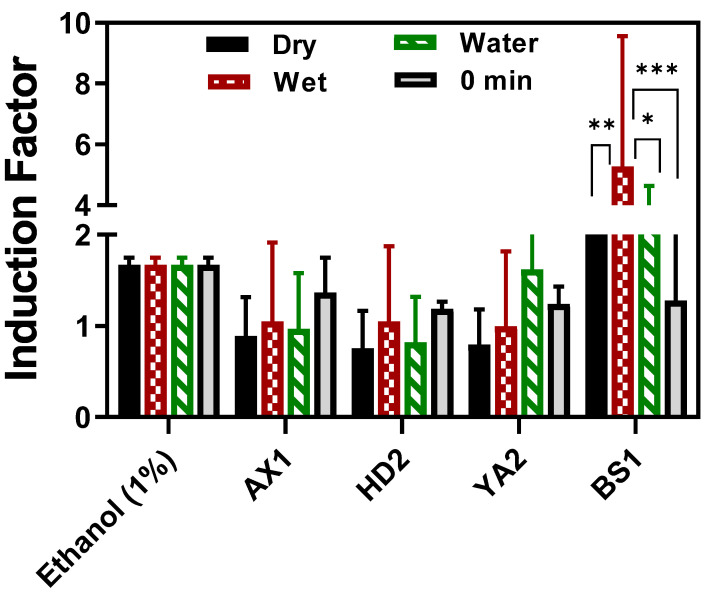
Fiber-optic-based reporter bioluminescent bacterial direct sediment toxicity response. The wet sediment and water samples were tested on-site, whereas the sediment extracts and dry sediment samples were tested in the laboratory after the samples were oven-dried, and the moisture content was determined. The results indicate that in several cases, especially in BS1 and YA2, the water samples yielded higher induction factors than the sediment, suggesting greater bioavailable toxicity in the aqueous phase. Ethanol was used as a positive control. The results for each location were statistically compared between dry and wet sediments and water collected from the same location (* *p* < 0.05, ** *p* < 0.01 *** *p* < 0.001). The results are expressed as the mean of replicate measurements, and the error bars represent the standard deviation (SD).

Toxicity measurements of the sediment and water samples were determined using our portable optical-fiber setup at four potentially contaminated sites in Israel, as shown in Figure 7. As can be seen, the established standard concentration of ethanol and the water sample of YA2 led to an induction factor higher than 1, while the wet, dry, and water samples displayed an induction factor value of less than 1. Since the reporter bacterial strain (*E. coli* TV106) harbors an inducible *lux* gene that is under the control of the grpE promoter, the activated shock response system, which is activated under conditions of cellular stress and metabolic changes, such as cytotoxic substances [19,21], different induction patterns were observed. When a stress stimulus is applied to the corresponding inducible lux bioreporter used in this study, its receptor proteins are activated, the signal inside the cell is transmitted to the promoter upstream of the *lux* genes, and their expression is induced. Several minutes after induction, luminosity rises, but when such a stimulus severely damages the cell, metabolism is impaired, and luminosity drops [22].

#### 3.1.2. The Laboratory-Based Toxicity Response and Calibration Curve

**Figure 8:**
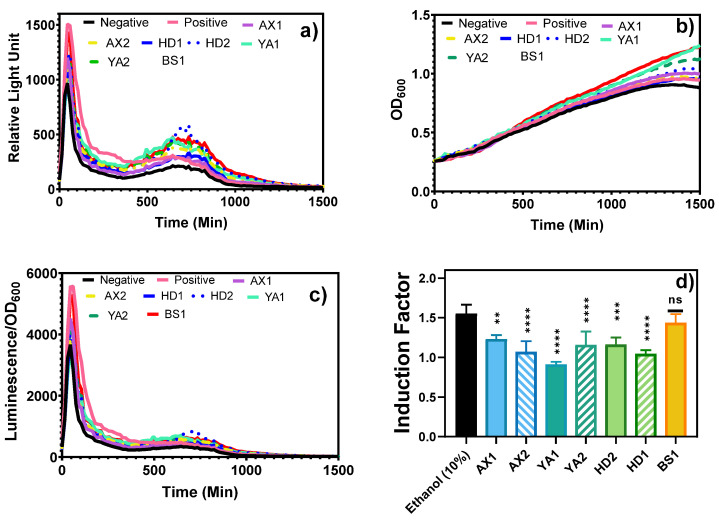
Bioluminescence and bacterial growth curves in response to bioavailable sediment extracts. (**a**) Kinetic curves of the bioluminescent response of the bioavailable toxicant obtained during the 24 h extraction. (**b**) Bacterial growth curve (OD_600_) during toxicity measurements in the presence of bioavailable sediment. (**c**) Kinetic curve normalized to OD_600_ at each time point. (**d**) The induction factor was calculated as described earlier as the ratio of induced bioluminescence to non-induced control. The results are reported as mean ± SD, n = 4. Statistical analysis revealed significant differences in the readings between different samples and ethanol at *p* < 0.05, 0.01, and 0.001, and ns represents no significant difference at *p* < 0.05. Results were considered statistically significant at *p* ≤ 0.05 (*), highly significant at *p* ≤ 0.01 (**), extremely significant at *p* ≤ 0.001 (***), and extremely significant at *p* ≤ 0.0001 (****), as indicated in the figure. Notably, the BS1 extracts triggered significantly higher normalized luminescence than the control (*p* < 0.001), indicating strong cytotoxic stress.

**Figure 9:**
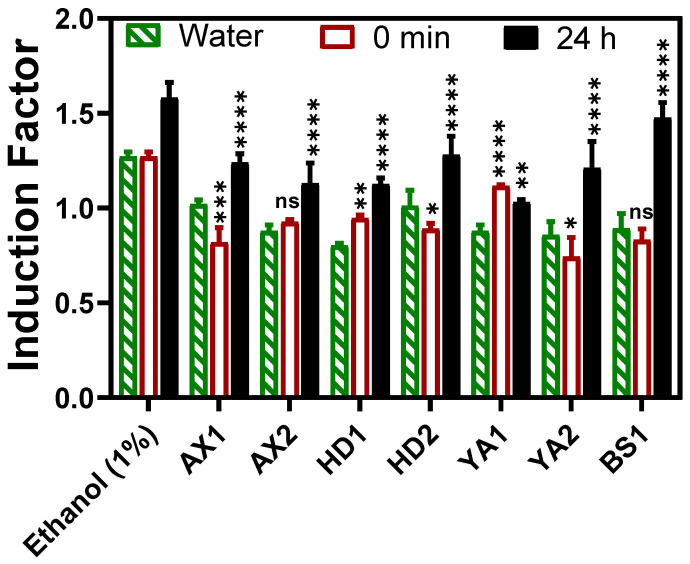
Bioluminescence responses of water and bioavailable sediment samples. The results showed a bioluminescent response to water samples and bioavailable toxicants prepared instantly or after 24 h of extraction at different times. The 24 h extracts generally produced stronger responses, likely due to the improved solubilization of hydrophobic toxicants. The results are reported as mean ± SD, n = 4. Statistical analysis revealed significant differences in the readings between 0 min and 24 h compared with water within each sample group at *p* < 0.05, 0.01, and 0.001, and ns represents no significant difference at *p* < 0.05. Results were considered statistically significant at *p* ≤ 0.05 (*), highly significant at *p* ≤ 0.01 (**), extremely significant at *p* ≤ 0.001 (***), and extremely significant at *p* ≤ 0.0001 (****), as indicated in the figure. A complete comparison is presented in Supplementary Table S3.

Furthermore, the fiber-optic-tested samples were brought to the laboratory for equipment-based toxicity testing of both the water and bioavailable sediment extracts (Figure 8 and Figure 9). The results showed that both treated and non-treated controls demonstrated similar growth curves, indicating that bioluminescence induction did not critically impair physiological processes or result in the death of the bioreporter (Figure 8b,c).

**Figure 10:**
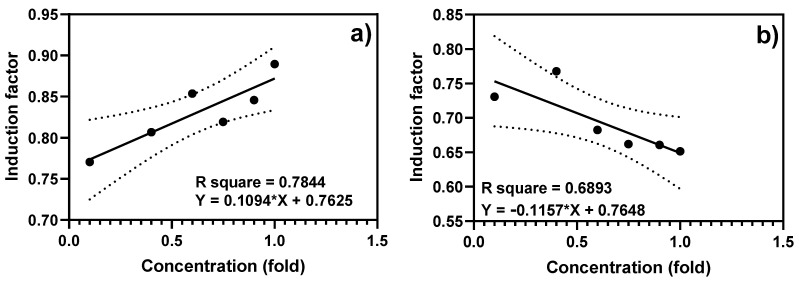
Calibration curve showing the dose-dependent bioluminescent response of the bioreporter to serially diluted sediment samples. (**a**) Luminescence intensity increased proportionally with sample concentration (sample from location BS1). (**b**) Bioluminescence decreased with increasing concentration, leading to a negative correlation (sample from location AX1). Linear regression lines (bold) were drawn on a linear scale, and the 95% confidence interval limits were the black dotted lines parallel to the regression lines, the black dots are the data points used in the plot. The equation and the *R*^2^ value for each regression line are shown. The result is presented as the mean ± SD of triplicate experiments.

To evaluate the dynamic range of the biosensor, a calibration curve was generated by exposing the system to serial dilutions of representative sediment and water samples in DDW. A dose-dependent change in bioluminescent intensity was observed, confirming the sensor’s responsiveness to varying toxicity levels. Importantly, depending on the nature and concentration of the toxicant, luminescence may increase or decrease, reflecting the activation or inhibition of bacterial metabolism [22]. As seen in Figure 10a, luminescence increased with sample concentration for the sediment samples collected from Beer Sheva River (BS1), while the sediment samples collected at Alexander River (AX1) showed a negative correlation, where the luminescence decreased with sample concentration, as shown in Figure 10b. These results support the biosensor’s potential for the semi-quantitative assessment of environmental toxicity.

#### 3.1.3. Viable Cell Quantification on the Fiber Probe

To determine the number of viable *E. coli* bioreporters immobilized on each fiber-optic probe, a colony-forming unit (CFU) analysis was performed. Individual alginate-coated probes were incubated in 50 mM sodium citrate buffer to dissolve the calcium alginate matrix and release encapsulated cells. The resulting suspension was serially diluted with sterile normal saline and plated onto LB agar. After 24 h of incubation at 37 °C, CFUs were counted (Supplementary Materials Figure S4). Across three biological replicates, the average number of viable cells per probe ranged between 3.3 × 10^9^ and 5 × 10^9^ CFU/mL per probe.

### 3.2. Conventional Analysis

Conventional chemical analyses were conducted to provide insights into the possible chemical composition of the tested samples using a combination of ICP-OES and LC-MS methodologies.

#### 3.2.1. ICP Analysis

Inductively coupled plasma optical emission spectroscopy (ICP-OES) was performed to qualitatively assess the elemental composition of water and sediment extracts from the tested sites. The presence or absence of key elements provides supportive evidence of contamination, complementing the biosensor data. The results of this analysis are summarized in Table 1.

#### 3.2.2. LC-MS Results and Analyses

**Figure 11:**
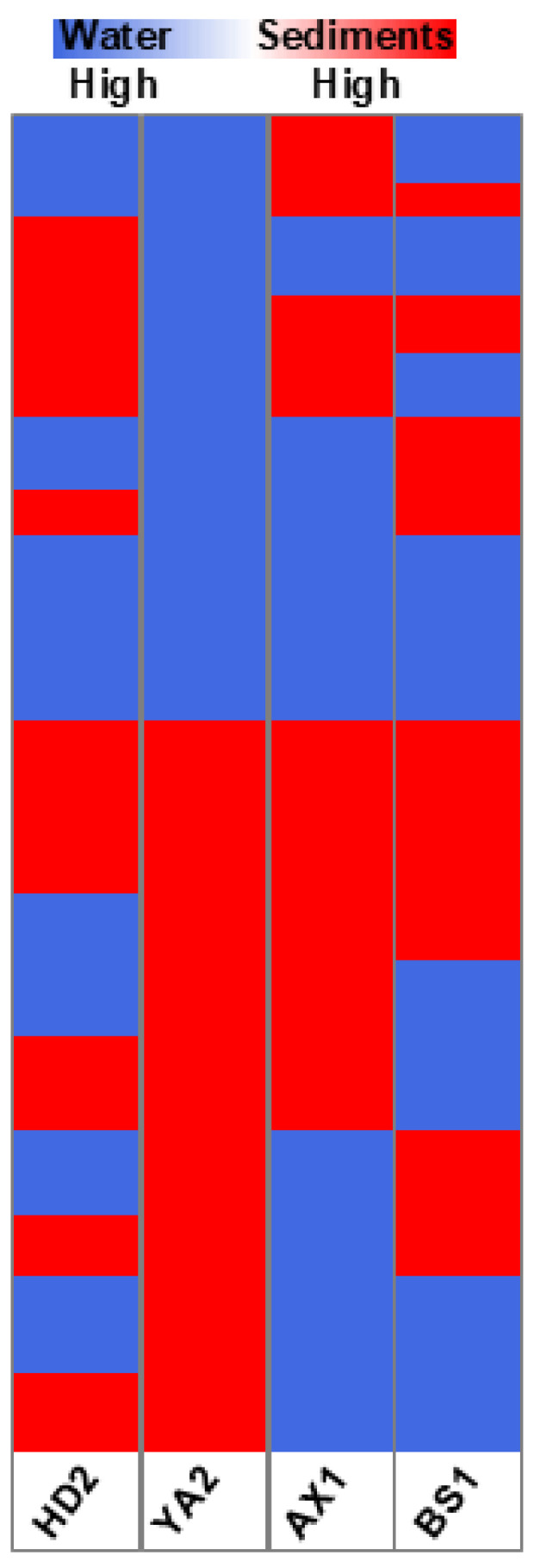
Heatmap of the comparative analysis of chemical composition of sediments versus pure water in four geographical locations (HD2, Hadera; YA2, Yarkon; AX1, Alexander; BS1, Beer Sheva). Blue color represents the chemicals found more abundantly in water; red color represents the chemicals found more abundantly in the sediment samples.

To explore the chemical composition of sediments and water in the four representative locations, we performed an untargeted metabolic analysis of bioavailable compounds extracted from sediments and those present in river water from the same locations, and compared these results with the chemical composition of pure water. The data obtained revealed 9644 chemical compounds released from sediments across all four locations, as well as a unique fingerprint for each region (Figure 11). Intriguingly, site AX1 showed the least specific fingerprint, while the three other sites were more enriched in unique compounds and at the same time shared more chemicals between themselves compared to AX1.

The analysis revealed a distinct partitioning of contaminants between the aqueous and solid phases, highlighting the differences in pollutant bioavailability and persistence. This comparative visualization aids in understanding the environmental distribution of contaminants and their potential impacts on aquatic ecosystems. 

**Figure 12:**
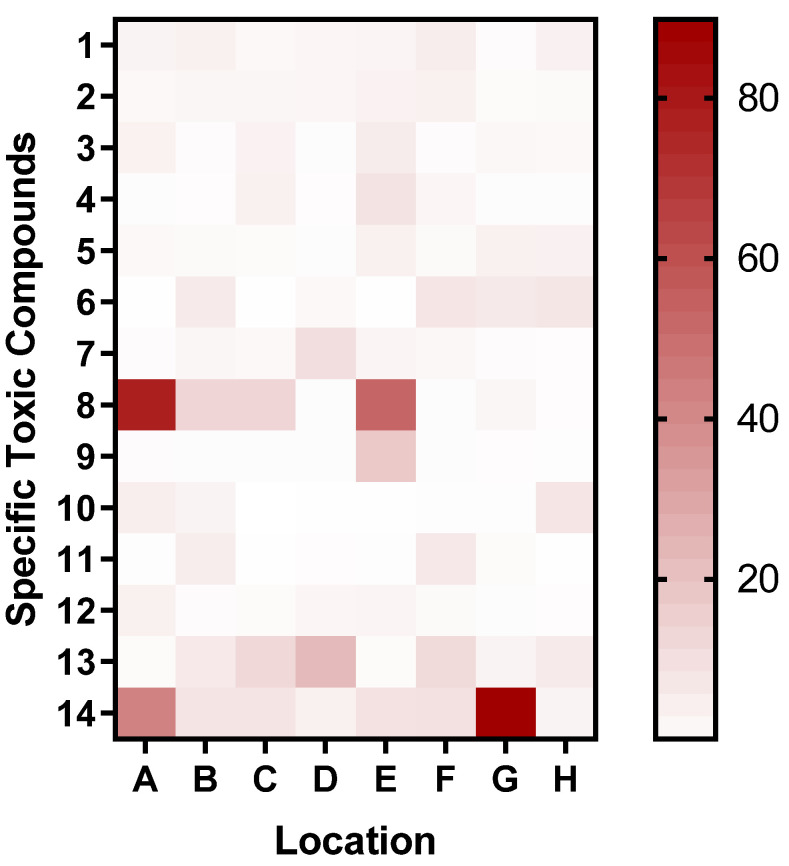
Heatmap of putatively annotated specific toxic compound distributions across various locations. The compounds identified from the LC-MS analysis and heatmap were generated as the ratio of the area under the peaks of the samples divided by that of the blank. Locations A, B, C, D, E, F, G, and H are the HD2 water, HD2 sediment, YA2 water, YA2 sediment, AX1 water, AX1 sediment, BS1 water, and BS1 sediment, respectively. BS1 samples contained high levels of triethanolamine and dichloromethane, which may explain their elevated bioluminescent induction factors. This highlights the site-specific chemical toxicity profiles.

Figure 12 presents a binarized heatmap comparing the chemical composition of the sediment and water samples collected from four different geographical locations (HD2, YA2, AX1, and BS1). The binarization approach simplifies visualization by categorizing compounds according to their relative abundance, highlighting whether a compound is predominantly present in sediment (shown in red) or water (shown in blue). This simplified color scale helps identify the partitioning behavior of bioavailable pollutants in environmental matrices.

BS1 and HD2 exhibited a broad diversity of compounds in both sediment and water fractions. This observation aligns with the relatively high bioluminescent induction factors reported for these sites (Figure 7). In contrast, AX1 showed fewer unique features, which may indicate a lower level of contamination or different compound solubilities and retention characteristics. The compound distribution patterns observed in Figure 10 emphasize the complex chemical landscape of these samples and justify the application of whole-cell biosensors, which measure the cumulative biological effects of complex contaminant mixtures instead of relying solely on chemical identification.

Considering the untargeted approaches to the analysis, we were unable to precisely identify the compounds. However, the use of exact m/z values detected for each chemical by high-resolution mass spectrometry allows them to be converted into potential chemical formulas. The results of this putative annotation suggest the distribution of specific toxic compounds across locations (Figure 12).

To facilitate interpretation of the compound-specific heatmap presented above, Table 2 provides the corresponding identification of each numbered toxicant. These compounds were annotated based on high-resolution mass spectrometry data using putative matching to public databases such as ChemSpider and mzCloud. The numeric labels in the heatmap reflect the entries listed in Table 2, allowing for easier cross-referencing and contextual analysis.

Table 3 presents the number of compounds in which each element was detected and its relative percentage of the total identified compounds. This comparison highlights the differences in element partitioning between the aqueous and sediment phases, indicating potential variations in bioavailability and environmental persistence.

## 4. Discussion

Bioavailability-based toxicity assessment has recently gained research interest because it allows a much more relevant representation of exposure to contaminants. This study presents a field-deployable bioluminescent-bacteria-based toxicity assessment of water and sediment collected from potentially contaminated sites in Israel. The bioreporter *E. coli* (TV1061) strain used in this study is an inducible *lux* biosensor with the lux genes under the control of a promoter activated in response to protein damage, producing a concentration-dependent signal output. The bioreporter was immobilized by adlayers of calcium alginate onto the tips of optical fibers, and the far end was connected to a photon-counting unit for real-time luminescent counting. This system enabled direct sediment toxicity assessment as calcium alginate provided a semi-permeable membrane that allows free diffusion of solutions containing small molecules, including contaminants, excludes larger particulate matter, and retains the bioreporter bacteria immobilized within the hydrogel.

All potentially contaminated sediment and water samples used in this study induced varying degrees of bioluminescence responses, as depicted in Figure 7. Dried and wet samples were analyzed by directly dipping the calcium alginate-immobilized bacteria optical fiber into the sediment-supplemented growth media (LB broth), and kinetic bioluminescent measurements were acquired. There was no statistically significant difference between the dried and wet sediment, or between the water samples collected at the same sample spot. Even when the direct sediment toxicity was compared with the extracted bioavailable toxicity (referred to as 0 min in Figure 7), there was no significant difference at *p* < 0.05. Thus, the direct sediment fiber-optic-based toxicity measurement setup in this study is invaluable for deployment in real-time toxicity assessment, offering an advantage in feasibility and a non-requirement of sample extraction steps, as the calcium alginate layer provided a semi-permeable barrier to particulate matter that could potentially interfere with luminescence measurement if other techniques, including luminometer measurement, were to be employed. The calcium alginate matrix serves as a semi-permeable barrier that allows toxicant diffusion while retaining immobilized bioreporters. However, it is possible that the matrix interacts with certain toxicants, particularly heavy metals, through ionic or electrostatic binding to the alginate carboxyl groups. Such interactions can potentially reduce the effective bacterial concentration. Although this was not directly quantified in the current study, future work will focus on systematically evaluating these effects using well-characterized toxicants. The advantages of hydrogel encapsulation are shown in Figure 5 and Figure 6. To the best of our knowledge, there are currently no available toxicity assessment methods that enable direct, real-time, on-site testing of untreated sediment samples. Conventional bioassays typically involve exposing model organisms to contaminated samples over several days, with endpoints such as mortality, reproduction, or growth inhibition. Although these assays provide ecologically relevant information, they are inherently time-consuming, require specialized laboratory facilities, and are not suitable for field deployment. In contrast, the fiber-optic whole-cell biosensor developed in this study offers a portable, rapid, and cost-effective solution for assessing general cytotoxicity in real time. By employing genetically engineered *E. coli* encapsulated in a calcium alginate matrix at the fiber tip, the system allowed direct exposure to complex environmental matrices without the need for prior sample extraction or pretreatment. The semi-permeable hydrogel layer selectively permits small molecules to diffuse into the bioreporter matrix while excluding particulates that may interfere with optical measurements.

Compared with instrumental chemical analyses (e.g., LC-MS and ICP-OES), which require extensive sample preparation and infrastructure, our biosensor offers biologically relevant toxicity readings in a field-deployable format. In addition, although microplate-based biosensors provide high sensitivity and stress-specific responses, they are limited by their dependence on controlled laboratory conditions and lack of spatial flexibility. The variability in toxicity levels observed across the tested sites in Israel underscores the urgent need for site-specific environmental management strategies. Although not suitable for continuous monitoring, the biosensor enables real-time measurements over a 24-h period with 5-min intervals. Owing to the limited viability of the reporter bacteria, this system is best used for periodic water quality assessments. Its portability, low cost, and minimal sample preparation make it ideal for on-site application. Similar challenges are faced by other countries in Europe and North America, where sediment toxicity testing is commonly incorporated into environmental regulations for both prospective and retrospective assessments [76]. Such sediment toxicity assessment practices and regulatory frameworks are missing in Africa, suggesting a potential research gap in this region [76]. Our findings highlight the potential of whole-cell biosensors in guiding targeted sustainability efforts by providing rapid on-site toxicity assessments that can be deployed in both low- and medium-income countries.

Complementary laboratory-based experiments were conducted using a microplate reader (Figure 8a and Figure 9) to determine the bioluminescent response of the bioavailable sediment extracts to corroborate the portable optical fiber setup. The bacterial growth curve was monitored by measuring the optical density at 600 nm (OD_600_) during the toxicity measurement to evaluate the relationship between the growth/density and the biosensor response of the reporter bacteria (Figure 8b). This further confirmed that bioluminescence induction did not lead to significant physiological harm or death of the bacteria. Bioluminescence was normalized to OD600 and used to calculate induction factors (Figure 8c,d). As can be seen, the BS1 sample produced a statistically similar (*p* < 0.05) bioluminescent response to that elicited by the standard inducer, showing the potential possibility of high contamination, as can be seen in Figure 7. Moreover, Beer Sheva River was once listed among highly polluted rivers, including the Na’aman, Zipori, Kishon, Taninim, Hadera (HD), Alexander (AX), Yarkon (YA), Ayalon, Soreq, Lachish, and Besor streams in Israel, with a range of pollutants, including nonpoint agricultural runoff, urban stormwater, and discharge from industrial sites, that can be found in many streams [18]. Although this study established a proof of concept for a portable, fiber-optic whole-cell biosensor capable of direct sediment and water toxicity measurements, some key performance parameters remain to be fully characterized. For instance, the sensor response time, defined as the time to reach peak bioluminescence following exposure, ranged from 400 to 900 min, as observed in the kinetic curves presented in Supplementary Figure S3. This variability was attributed to the nature of the toxicant and its mechanism of action in activating the grpE-regulated lux reporter system. While this response profile is suitable for semi-rapid toxicity assessments, further optimization is required for high-throughput or time-critical applications. Moreover, comprehensive analyses of the detection limits, specificity, and reproducibility are ongoing in our laboratory. These include controlled exposure to individual toxicants across various chemical classes and the use of orthogonal detection platforms for validation. While preliminary results indicate promising sensitivity and target responsiveness, definitive metrics for the limit of detection (LOD) and inter-assay variability will be reported in future publications. We recognize that such quantitative parameters are essential for the regulatory acceptance and widespread adoption of this biosensing platform in environmental monitoring frameworks.

Furthermore, chemical analyses, such as ICP-OES, revealed the presence of multiple elements in the water and sediment samples, including calcium, magnesium, potassium, sodium, and sulfur, which are often associated with industrial or agricultural runoff [18]. Notably, untargeted LC-MS analysis revealed a diverse and site-specific chemical composition, with over 9644 compounds detected across the four representative sampling locations. Considering the untargeted approach to the analysis, we were unable to precisely identify the compounds. However, we used the exact m/z values detected for each chemical, using a high-resolution instrument, to convert them into potential molecular formulas. The results of this conversion suggested a high presence of sulfur-, chlorine-, and phosphorus-containing compounds, particularly in the river water of the collected samples, compared to the sediment extracts.

Next, we performed putative annotation for compounds found to be more abundant in sediment samples by screening the detected m/z values versus public high-resolution MS databases (ChemSpider and HMDB). This unraveled some interesting potential hints, including Adenine, Guanine, Uracil, Cytosine, and Thymine, suggesting the presence of fossils in the sediments of all four locations, although confirmation would require a targeted metabolomics approach. Additionally, LC-MS analysis detected several potentially toxic compounds at the tested sites, such as Ethephon [50,51,52], N, N-Dimethylacetamide [56,57,58], Diethanolamine, Phenylethyl alcohol, and dichloromethane (Table 2). These compounds provide clues for the increasing I.F obtained from the biosensor. Although untargeted LC-MS profiling provides valuable insight into the chemical complexity of samples, the ability to correlate individual compounds to bioluminescent responses remains limited due to the unknown nature and co-occurrence of multiple toxicants. However, tentative associations can be made. For instance, the high relative abundance of triethanolamine in the BS1 water sample coincided with a high induction factor in the biosensor assay, suggesting a potential link. To establish quantitative structure–activity relationships, future work will incorporate targeted validation with pure standards and multivariate statistical analyses to correlate the chemical composition with biosensor responses. While these traditional methods (such as LC-MS and ICP-OES) offer the precise quantification of specific contaminants, they fail to assess the bioavailable fraction of contaminants and their synergistic effects on biota. Our biosensor addresses this gap by providing rapid on-site bioavailable toxicity data on living systems. Although the current biosensor detects general cytotoxicity, it does not distinguish between specific types of toxicants. Different contaminants, such as heavy metals and organic pollutants, may trigger distinct magnitudes of bioluminescence owing to their varying mechanisms of action [77]. However, the biosensor provides a reliable assessment of the overall toxic burden using an induction factor. Ongoing work is focused on incorporating multiple stress-specific bioreporters to enable the differentiation between toxicant classes in complex mixtures.

The resolution of the biosensor measurements can be interpreted across multiple contexts. Temporally, the system records the luminescent output every 5 min over a 24-h period, allowing high-resolution monitoring of signal dynamics, including onset, peak, and decay patterns. Biologically, the biosensor demonstrates a reproducible dose-dependent response to increasing toxicant concentration, which supports its semi-quantitative use in toxicity screening (Figure 10). Chemically, while the sensor does not distinguish between specific toxicants, it reliably indicates the presence of biologically active compounds. Future adaptations incorporating multiplexed bioreporters or differential stress pathways may enhance the sensor’s resolution in distinguishing among toxicant classes.

Despite these strengths, this study had some limitations. Although the fiber-optic biosensor was portable, the current setup requires manual operation, careful fiber handling, and a degree of technical proficiency. Black-box housing and heating mats, although functional, are not optimized for rugged or long-term outdoor use. Future improvements could include automation, wireless data logging, user-friendly interfaces, and the miniaturization of optical and electronic components. Additionally, although the use of a general stress-responsive promoter allows broad toxicity detection, employing a panel of bioreporters targeting specific stress pathways or contaminant classes could expand the sensing capabilities of the system.

## 5. Conclusions

This study demonstrates the development of a portable, whole-cell fiber-optic biosensor using modified *E. coli* to measure sediment and water toxicity across contaminated sites in Israel. Our findings show that this biosensor provides a rapid and cost-effective method for assessing environmental toxicity, bridging the gap between chemical analysis and bioassays. The calcium alginate matrix for immobilizing reporter bacteria on fiber-optic tips has proven effective in protecting bioreporters while allowing toxicant passage and enabling accurate on-site assessment without complex sample preparation.

The deployment of the biosensor revealed variations in bacterial bioluminescent responses, reflecting the diverse toxicants in the environment. This variability demonstrates the sensitivity of the biosensor for monitoring environmental pollution and ecosystem protection.

This study identified challenges regarding the portability of a system’s field operations. Future research should focus on improving the biosensor design for remote environmental settings and expanding bioreporter strains to include those responsive to specific contaminants, thus enhancing their monitoring utility.

## Figures and Tables

**Table 1:** ICP results for different samples analyzed in this study. + and − indicate that the element was detected or not detected, respectively.

Element	HD1	AX2	YA1	YA2
Ag	−	−	−	−
Al	−	−	−	−
B	−	+	−	−
Ba	−	−	−	−
Bi	−	−	−	−
Ca	+	−	+	−
Cd	−	−	−	−
Co	−	−	−	−
Cr	−	−	−	−
Cu	−	−	−	−
Fe	−	−	−	−
Ga	−	−	−	−
K	+	+	+	+
Li	−	−	−	−
Mg	+	+	+	−
Mn	−	−	−	−
Na	+	+	+	+
Ni	−	−	−	−
Pb	−	−	−	−
Sr	−	−	−	−
Zn	−	−	−	−
S	+	+	+	+
P	−	−	−	−

**Table 2:** The identification of the different toxic compounds listed in the heatmap above.

Number	Name	References
1	Bis(2-ethylhexyl) amine	[32]
2	Chlorphentermine	[33,34,35]
3	Cyclohexylamine	[36,37,38,39]
4	Dichloromethane	[40,41,42]
5	Dicyclohexylamine	[43,44,45]
6	Diethanolamine	[46,47,48,49]
7	Ethephon	[50,51,52]
8	MDMA	[53,54,55]
9	N,N-Dimethylacetamide	[56,57,58]
10	N-ethylmaleimide	[59,60,61]
11	o-Toluidine	[62,63,64,65]
12	Phentermine	[66,67,68,69]
13	Phenylethyl alcohol	[70,71,72]
14	Triethanolamine	[46,48,73,74,75]

**Table 3:** Elemental distribution in water and sediment samples based on LC-MS analysis.

Element	Compounds in Water	Proportion in Water (%)	Compounds in Sediment	Proportion in Sediment (%)
Sulfur	299	40.13	154	14.41
Chlorine	279	37.45	35	3.27
Bromine	110	14.77	7	0.65
Fluorine	3	0.4	6	0.56
Iodine	2	0.27	1	0.09
Selenium	1	0.13	1	0.09
Indium	1	0.13	0	0
Phosphorus	422	56.64	198	18.52
Nitrogen	497	66.71	862	80.64

## Data Availability

The original results presented in this study are included in the article and Supplementary Materials, and further inquiries in terms of data can be found in our records in footprints (https://footprints-b291f.web.app/); authorization for access may be granted by the corresponding author (rsmarks@bgu.ac.il).

## Supplementary Materials

The following supporting information can be downloaded at https://www.mdpi.com/article/10.3390/bios15070404/s1. **Figure S1:** The appearance of the adlayers of alginate onto the fiber optic tips before and after measurement.; **Figure S2:** Direct testing using the Fiber Optics Black Box at Beersheba Stream; **Figure S3**: Representative fiber optic measurements; **Figure S4.** Representative CFU enumeration plates showing serial dilutions of bacteria released from individual fiber-optic probes. **Table S1:** Sample collection sites’ coordinates; **Table S2:** Sediments’ Moisture Content; **Table S3:** Tukey’s multiple comparison (Statistical Analysis of Figure 9).
