# Supplementary material for: Whole-Cell Fiber-Optic Biosensor for Real-Time, On-Site Sediment and Water Toxicity Assessment: Applications at Contaminated Sites Across Israel
Source: Biosensors (Basel). 2025 Jun 22;15(7):404. doi: 10.3390/bios15070404 (PMC12293957; doi:10.3390/bios15070404)
Supplement: Supplementary file 1 [file biosensors-15-00404-s001.zip › biosensors-3640350-supplementary.pdf]

Supplementary materials

1. Methodology

Table S1: Sample collection sites' coordinates

Site	Sample	Coordinates
Hadera Stream	HD1	32°27'51"N 34°53'11"E
	HD2	32°27'53"N 34°53'32"E
Alexander River	AX1	32°23'38"N 34°52'11"E
	AX2	32°23'46"N 34°51'57"E
Yarkon River	YA1	32°05'58"N 34°47'56"E
	YA2	32°06'03"N 34°46'40"E
Beersheba Stream	BS1	31°14'09"N 34°47'37"E

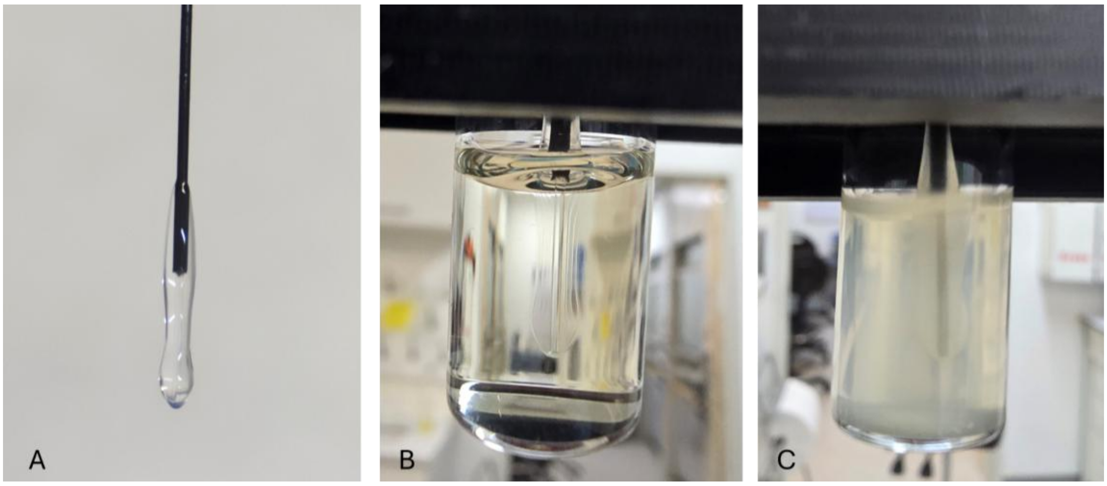

Figure S1: The appearance of the adlayers of alginate onto the fiber optic tips before and after measurement. A) Probe prior immersion in water sample; B) Probe immersed in clear water sample, LB broth, and $CaCl_2$ solution; C) Turbid solution with the probe following measurement.

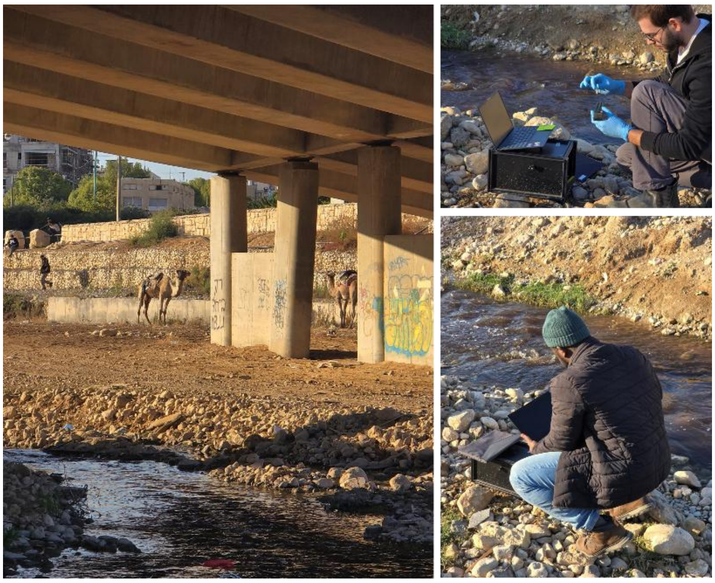

Figure S2: Direct testing using the Fiber Optics Black Box at Beersheba Stream.

Results

Table S2: Sediments' Moisture Content

Sample ID	Moisture Content (%)
HD1	15.18
HD2	36.92
AX1	11.30
AX2	18.72
YA1	25.35

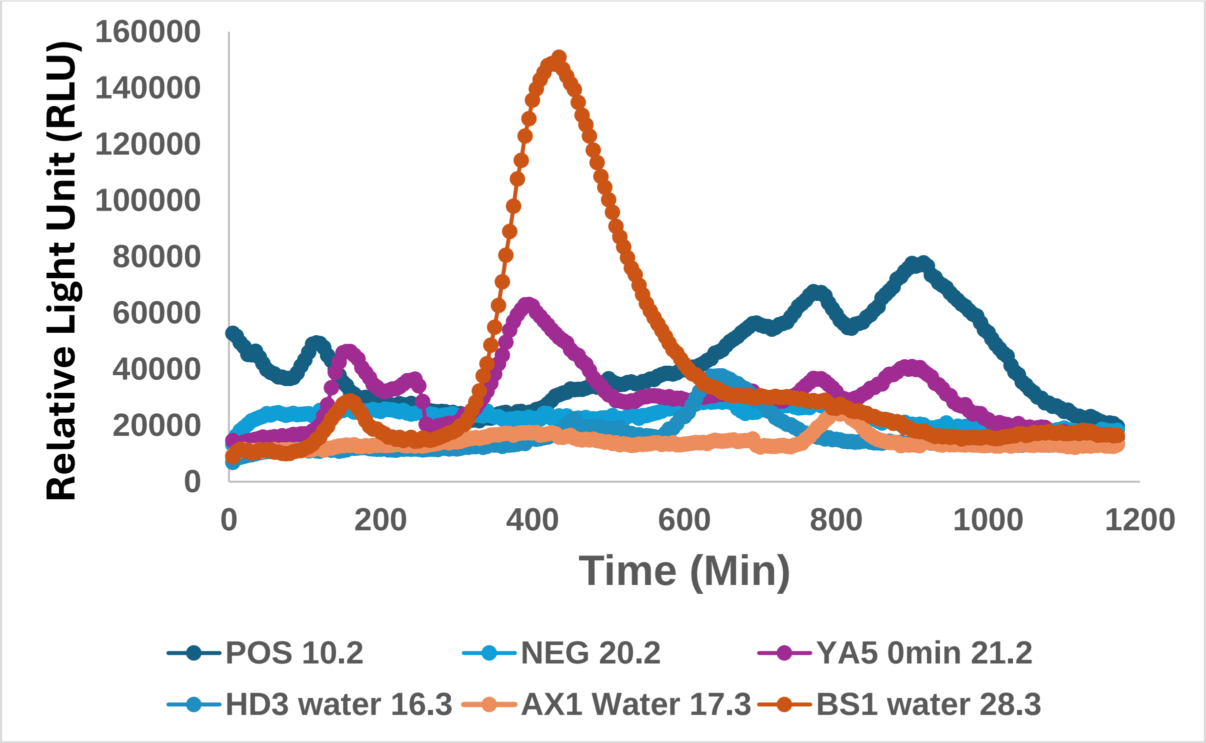

Figure S3: Representative fiber optic measurements

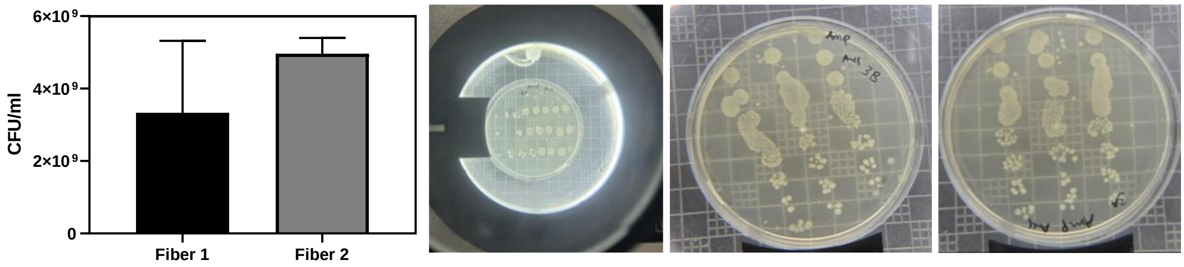

Figure S4. Representative CFU enumeration plates showing serial dilutions of bacteria released from individual fiber-optic probes. The probe-encapsulated bioreporters were dissolved in sodium citrate buffered, diluted with normal saline, and plated on LB agar to assess viable cell numbers. Colony growth confirmed the presence of approximately 3.3×10^9 to 5×10^9 CFU/ml per probe.

Table S3: Tukey's multiple comparison (Statistical Analysis of Figure 9)

**Statistical Analysis of Figure 9**					
Within each row, compare columns (simple effects within rows)					
Number of families	8				
Number of comparisons per family	3				
Alpha	0.05				
Tukey's multiple comparisons test	Mean Diff.	95.00% CI of diff.	Significant?	Summary	Adjusted P Value
Ethanol (1%)					
24 h vs. 0 min	0.3095	0.1990 to 0.4199	Yes	****	<0.0001
24 h vs. Water	0.3095	0.1990 to 0.4199	Yes	****	<0.0001
0 min vs. Water	0	-0.1104 to 0.1104	No	ns	>0.9999
AX1					
24 h vs. 0 min	0.4203	0.3098 to 0.5307	Yes	****	<0.0001
24 h vs. Water	0.2189	0.1084 to 0.3293	Yes	****	<0.0001
0 min vs. Water	-0.2014	-0.3118 to -0.09096	Yes	***	0.0001
AX2					
24 h vs. 0 min	0.2044	0.09391 to 0.3148	Yes	****	<0.0001
24 h vs. Water	0.2516	0.1411 to 0.3620	Yes	****	<0.0001
0 min vs. Water	0.04721	-0.06323 to 0.1577	No	ns	0.5649
HD1					
24 h vs. 0 min	0.1776	0.06712 to 0.2880	Yes	***	0.0007
24 h vs. Water	0.3222	0.2118 to 0.4327	Yes	****	<0.0001
0 min vs. Water	0.1447	0.03424 to 0.2551	Yes	**	0.0069
HD2					
24 h vs. 0 min	0.3898	0.2794 to 0.5003	Yes	****	<0.0001
24 h vs. Water	0.2683	0.1579 to 0.3788	Yes	****	<0.0001
0 min vs. Water	-0.1215	-0.2320 to -0.01108	Yes	*	0.0276
YA1					
24 h vs. 0 min	-0.08501	-0.1955 to 0.02543	No	ns	0.1633
24 h vs. Water	0.1523	0.04185 to 0.2627	Yes	**	0.0042
0 min vs. Water	0.2373	0.1269 to 0.3477	Yes	****	<0.0001
YA2					
24 h vs. 0 min	0.4675	0.3570 to 0.5779	Yes	****	<0.0001
24 h vs. Water	0.3533	0.2429 to 0.4637	Yes	****	<0.0001
0 min vs. Water	-0.1142	-0.2246 to -0.003720	Yes	*	0.0412
BS1					
24 h vs. 0 min	0.6457	0.5353 to 0.7562	Yes	****	<0.0001
24 h vs. Water	0.5847	0.4743 to 0.6952	Yes	****	<0.0001
0 min vs. Water	-0.06103	-0.1715 to 0.04941	No	ns	0.3875
